# Modified formulas for calculation of encephalization: quotient in dogs

**DOI:** 10.1186/s13104-021-05638-0

**Published:** 2021-06-03

**Authors:** Saganuwan Alhaji Saganuwan

**Affiliations:** Department of Veterinary Pharmacology and Toxicology, College of Veterinary Medicine, Federal University of Agriculture, Benue State, P.M.B. 2373, Makurdi, Nigeria

**Keywords:** German shepherd, Allometry, Encephalization quotient, Intelligence

## Abstract

**Objective:**

Dogs are a breed of animals that play important roles in security service, companionship, hunting, guard, work and models of research for application in humans. Intelligence is the key factor to success in life, most especially for dogs that are used for security purposes at the airports, seaports, public places, houses, schools and farms. However, it has been reported that there is correlation between intelligence, body weight, height and craniometry in human. In view of this, literatures were searched on body weight, height and body surface areas of ten dogs with intent to determining their comparative level of intelligence using encephalization quotient.

**Results:**

Findings revealed that dogs have relationship of brain allometry with human as proven by encephalization quotient $\left( {{\text{EQ}}} \right)\, = \,{\text{Brain Mass}}/0.{14}\, \times \,{\text{Body weight}}^{{0.{528}}} ,{\text{ Brain Mass}}/0.{12}\, \times \,{\text{Body Weight}}^{{0.{66}}}$

and Brain Mass (E)  =  kpβ, where p is the body weight; k  =  0.14 and β = 0.528, respectively. Saganuwa’s formula yielded better results as compared with the other formulas. Dogs with body surface area (BSA), weight and height similar to that of human are the most intelligent. Doberman pinscher is the most intelligent followed by German shepherd, Labrador retriever, Golden retriever, respectively.

## Introduction

The neural basis of human intelligence in relation to brain weight and head circumference has been identified by computed tomography (CT) and magnetic resonance imaging (MRI). The value range of 0–0.6 with verbal ability correlating with cerebral volume has been reported for each hemi-sphere in women, and in right handed men accounting for 36% of the variation in verbal intelligence [1]. Body temperature, food digestion, and phylogeny do not support the scaling of basal metabolic rate (BMR) to 3/4, but support the scaling to 2/3 [2]. Lack of a single exponent model, suggests that there is no universal accepted allometry [3]. Similar objects exhibit isometry, whereas geometrically dissimilar objects exhibit allometry. Brain weight is an index of intelligence [4], low birth weight is related to intelligence in 3–5-year-old children [5] and variation in brain size is related to intellectual achievement [6]. Numerical comparison of relative brain to body size is called encephalization quotient (EQ) [7]. The EQs for pig and sheep (0.6), giraffe (0.7), Bactrian camel (0.8), Llama and guinea pig (0.9), European cat (1.1), dog (1.2), vicuna (1.4), ring-tailed lemur (1.5), gorilla (1.4–1.7), fox (1.6), Asian elephant (2.3), chimpanzee (2.2.–2.5) and human (7.3–7.7), respectively have been established [7–10].The efficiency of structural organization of brain, could be an important biological basis for intelligence [11]. Metabolic processes and brain size share some relationship with body size across mammals. Hence, lean body mass is the more appropriate scaling parameter for comparing brain size across species [12]. The EQ for dolphin (5.3) and monkey (4.8) suggest that intelligence may depend on the amount of brain nerves, brain’s menial chores and brain size [13]. Results from evaluations of placental-brain size cannot be applied generally to mammals [14]. The study of human evolution related large brains to increased capacity of expertise not intelligence quotient [15]. Expansion and differentiation of neocortex increase brain size and complex function of the brain [16], carnivores having intermediate values of brain size [17]. Human brain volume, grey matter, white matter, cortical thickness, cortical convolution and neural efficiency are used to measure intelligence [18]. Since different formulas used to calculate EQ provided different values; there is need for modification of the formulas with a view to providing formula that would provide efficient EQ.

## Main text

### Materials and methods

All the mathematical equations used in the present study have relationship between brain mass and body height of man. The formulas are presented as follow: Brain Mass  = 920 g(± 113) + 2.70 (± 0.65)  ×  body height of man; Brain Mass = 748 g(± 104) + 3.10 (± 0.64)  ×  body height of woman [19]. Animal EQ is calculated using the formula,

$$\frac{Brain \,Mass}{{0.12 \times Body \,weight^{{{\raise0.7ex\hbox{$2$} \!\mathord{\left/ {\vphantom {2 3}}\right.\kern-\nulldelimiterspace} \!\lower0.7ex\hbox{$3$}}}} }}$$ [17], modified $$ {\text{as}} \,\,(\frac{Brain \,Mass}{{0.14 \times Body\, weight^{0.528} }}$$) and used for the present study. Majority of animals are assumed to have an EQ of 1. Therefore a value greater than 1, may suggest higher than average intelligence [7]. However, percent body fat (% bf)  =  0.339 + 2.942 (logWT) [12], where WT is body weight, should be considered when the dogs are either obese or over weight. The formula for linking hominid skull volume to brain volume is log_10_ (B)  =  3.015 + 0.986 log_10_(C), where B (total brain size in mm^3^) and C (internal cranial capacity in cubic millimeter) is expressed as y = 0.39x^0.27^, where 0.39 (integral constant), X (body weight mean) and 0.27 (allometric exponent).These parameters are potentially associated with intraspecies ratio of body weight and body weight means [20]. Also brain weight is calculated as E = kρβ where E is the brain weight, ρ is the body weight, k and β are determined from log–log plot of brain weight to body weight. Log k is the log E intercept and β is the slope [17]. Whereas k  =  0.18 and β  =  0.66 respectively [21]. However k  =  0.16 and β  =  0.67 have also been reported [17]. The intelligence of dogs was classified according to Coren [22] in decreasing order as follows: Border collie, Standard Poodle, German shepherd, and Golden retriever, Doberman pinscher, Shetland, Labrador retriever, Papillion, Rottweiler and Australian Cattle Dog used in the present study. Their body weight, body surface area, height, k  =  0.14 and β  =  0.528 were applied as reported by Saganuwan [23]. Total brain volume and age of the dogs were calculated using the formulas TBV  = 182.3 + 0.7  ×  Cranial Capacity and TBV = 396.5 X Age + 0.7 ×  CC respectively [24]. One-year mature-senior dog is equivalent to 13.125 year human. Also the age of dog based on epigenetics is calculated thus: $${\text{Dog Age}}\, = \,{\text{A}}\, \times \,{\text{ln }}\left( {\text{Human Age}} \right)\, + \,{\text{B}}$$, whereas A and B are coefficients estimated by bootstrapping an equal number of both humans and dogs [25].

## Results

The body weight, brain weight, body surface area, height and encephalization quotient of the ten dogs are presented in Table 1. The calculated brain weights for all the dogs using von Bronin’s formula were higher than the weights yielded by Jerison’s and Saganuwan’s formulas. Saganuwan’s formula yielded highest EQ for Border collie (2.3), Standard Poodle (2.2), German Sheperd (3.1), Golden Retriever (2.8), Doberman pinscher (3.1), Labrador retriever (2.9) and Australian Cattle Dog (2.7) as compared to the other formulas. However von Bronin’s formula yielded highest EQ for Rottweiler (2.5) as Jerison’s formula yielded highest EQ for Shetland Sheep Dog (1.4) and Papilon (1.2). German Shepherd had highest total brain size (TBS)/internal cranial capacity (ICC) ratio (1.4) followed by Labrador Retriever, Doberman pinscher, Golden retriever (1.3), Border collie, Standard Poodle, Papilon (1.2) and Shetland Sheep Dog (1.1), respectively. The calculated ICC parameter showed that Rottweiler and German shepherd scored highest, 11 (Table 1).

**Table 1:** Body weight, brain weight, body surface area, height and encephalization quotient of the reported intelligent dogs

Breed of dog	Dog age (year)	HEA (year)	BW (kg)	BSA (m^2^)	Height (m)	BMI	TBV (cm^3^)	Measurement of encephalization quotient						Ranking	ICC	TBS	$\frac{TBC}{ICC}$	Responsibilities
								EvB	ES	EJ	EQJ	EQvB	EQS					
Border collie	4.7	61.2	23.0	1.12	0.78	20.5	188.6	2.7	1.7	1.8	1.8	1.9	2.3^a^	7th	9	10.8	1.2	Herding
Standard Poodle	4.7	61.2	22.0	0.62	0.76	35.5	188.6	2.6	1.6	1.8	1.9	1.9	2.2^a^	8th	9	10.8	1.2	Bird retrieval
German shepherd	4.7	61.6	40.0	1.13	1.30	35.4	190	4.8	3.0	3.2	2.3	2.1	3.1^a^	2nd	11	15.4	1.4	Working
Golden retriever	4.7	61.4	34.0	1.22	1.00	27.9	189.3	4.0	2.5	2.7	2.1	2.2	2.8^a^	4th	10	12.9	1.3	Bird retrieval
Doberman pinscher	4.7	61.4	40.6	1.42	1.18	28.6	189.3	4.8	3.0	3.3	2.3	2.3	3.1^a^	1st	10	12.9	1.3	Guarding, protecting, war, work
Shetland Sheepdog	4.6	60.8	7.0	0.82	0.35	8.5	187.2	0.8	0.5	0.6	1.4^a^	1.2	1.3	9th	7	8.0	1.1	Herding, sport
Labrador retriever	4.7	61.4	36.0	1.14	1.00	31.6	189.3	4.3	2.7	2.9	2.2	2.2	2.9^a^	3rd	10	12.9	1.3	Bird retrieval
Papilon	4.6	60.6	4.5	0.23	0.56	19.6	186.5	0.5	0.3	0.4	1.2^a^	1.0	1.0	10th	6	6.9	1.2	Rat killing
Rottweiler	4.7	61.6	50.0	1.36	1.30	36.8	190	5.9	3.7	4.02	2.4	2.5^a^	1.8	6th	11	15.4	1.4	Guarding, working, military
Australian Cattle Dog	4.7	61.4	33.0	0.96	0.87	34.4	189.3	3.9	2.4	2.7	2.2	2.2	2.7^a^	5th	10	12.9	1.3	Herding

*BW* body weight; *BSA* body surface area; *E* brain mass; *EQ* Encephalization quotient; *TBV* total brain volume; *ICC* internal cranial capacity; *J* Jerison; *vB* von Bronin; *S* Saganuwan; *TBS* total brain size; *TBC* total brain volume; *HEA* human equivalent age; *BMI* body mass index

^a^Highest EQ in the row

## Discussion

The intelligence quotient of 1.0–3.1 reported for the dogs used in the present study disagrees with the report indicating that, the EQ for dogs was 1.2 [7–10]. EQs calculated from Saganuwan’s formula developed for both dog and human, shows that Doberman pinscher has the highest EQ, hence, considered the most intelligent followed by German shepherd, Labrador retriever, Golden retriever, Australian Cattle Dog, Rottweiler, Border collie, Standard Poodle, Shetland Sheep Dog and Papilon, respectively. The EQ  >  1 indicates larger brain, EQ  =  1(average) and smaller (EQ  <  1), respectively [26]. Each of the formulas used to calculate EQ yields different values, but the formula developed by Saganuwan may be more reliable. The TBS/ICC value higher than 1 and EQ value of 1.0–3.1 show that even Papilon is intelligent. Intelligence, learning, awareness and the welfare are closely related. Self-aware animals should be able to deduce mental states of other animals [27]. This is classified as a form of complex learning [28], signifying that level of intelligence is directly proportional to level of awareness [7]. However, awareness is a state that comprises conscious thought and unanxious responses [29]. Learning and awareness are connected with environment, past experiences, relationships between old and new information, and action to produce a positive outcome [30]. Acquisition of neutral information with no immediate effects on behavior is known as latency [31], seen between a naive and experienced animal [7]. However dog’s intelligence is of three components; instinctive intelligence, adaptive intelligence as well as working and obedience intelligence [22].Therefore the intelligence of dogs may be classified according to their functions as working (e.g., Doberman pinscher, German shepherd, Border collie), hunting (Labrador retriever, Golden retriever), companion (Standard Poodle) and toying (Papilon) intelligence, respectively [23]. Nutrition, genetics, environment, diseased condition inter alia, may affect EQ. However, study on evolution of encephalization quotient revealed that carnivore and other mammals showed abrupt increase in median log-encephalization quotients, indicating higher brain volume relative to body mass, at the end-midcene, but gradual increases in the variance of log EQs. By akaike information criterion, evolution of camid encephalization proposed plesiomorphic and apomorphic allometries [32]. Hence increased camid encephalization coincides with reorganization of the brain, which reflects complex social behaviour overtime [33]. The reported value of EQ (0.4) for African grass cutter [34] shows that the animal’s level of intelligence is quite low as compared to that of dog, which may allow easy predation by dog on the grass cutter. In another development, the therapeutic implication of EQ  >  1 is that, central nervous system acting drug, which has fulfilled the condition of cerebrospinal fluid (CSF) penetration, may be accumulated more in the brain tissues of dogs with higher EQ. This is because the larger the volume of the brain tissue, the higher the apparent volume of distribution of the drug. Relative reduction in brain size could increase the chance of obesity [35], and obese dog may be relatively dull. Hence fat could affect calculated value of encephalization quotient [36]. The brain weight could be an indicator of cognitive capacity development [37]. Therefore cognition is related to neurobiology and ecology [38]. More encephalized species have larger promontorial canal relative to transverse foramina, thereby increasing endocranial volume [39]. DNA methylation is a diagnostic tool for physiological aging in dogs [40], as observed in the present study, comprising dogs of 4.6 and 4.7 years of age, that may not be vulnerable to hippocampal atrophy, which could cause cognitive dysfunction in dogs of age  ≥  9 years. Hippocampal atrophy is a neurodegenerative disease in dogs similar to Alzheimer’s disease of human [41]. Dogs that have higher body weight relative to height, such as Doberman pinscher, German shepherd, Labrador retriever and Golden retriever rated first to fourth intelligent agree with the report, indicating that there is strong association between physical attractiveness and intelligence [42–44]. The difference in intelligence of the dogs in the present context may be due to significant anatomical variation among the breeds [45], suggesting that dogs have large number of neurons in the cerebral cortex [46]. Hence they are voice-sensitive [47]. Also temporal cortex activation is highly functional in perception of human faces by dogs [48], which is dependent on colour vision, sensitivity to light, visual acuity and cognition capacity [49]. Therefore animality is a threshold between human being and other animals [50].

## Conclusion

Body weight, height and body surface area can be used to estimate encephalization qotient of dogs, which can vary according to formulas, nutrition, environment and diseased conditions. The calculated parameters showed that Doberman pinscher is the most intelligent followed by German shepherd, Labrador retriever, Golden retriever, Australian Cattle Dog, Rottweiler, Border collie, Standard Poodle, Shetland Sheep Dog and Papilon, respectively.

## Limitations

The calculations were based on different formulas generated for different or similar purposes in dog and human. Intelligence in dogs and human is used for different assignments even within canine species. More so difference in physiology, anatomy and biochemistry count for differences in the calculated values.

## Data Availability

All data generated or analyzed are included in this published article.

## Abbreviations

P: Body weight
K: 0.14
β: 0.528
EQ: Encephalization quotient
